# Maternal adverse childhood experiences on child growth and development in rural Pakistan: An observational cohort study

**DOI:** 10.1371/journal.pgph.0001669

**Published:** 2023-10-25

**Authors:** Esther O. Chung, Elissa Scherer, Katherine LeMasters, Lisa Bates, Ashley Hagaman, Brooke S. Staley, Lauren C. Zalla, Siham Sikander, Joanna Maselko

**Affiliations:** 1 Department of Epidemiology, Gillings School of Global Public Health, University of North Carolina at Chapel Hill, Chapel Hill, North Carolina, United States of America; 2 Carolina Population Center, University of North Carolina at Chapel Hill, Chapel Hill, North Carolina, United States of America; 3 Department of Epidemiology, Mailman School of Public Health, Columbia University, New York, New York, United States of America; 4 Department of Social and Behavioral Sciences, Yale School of Public Health, New Haven, Connecticut, United States of America; 5 Center for Methods in Implementation and Prevention Science, Yale School of Public Health, New Haven, Connecticut, United States of America; 6 Department of Primary Care and Mental Health, University of Liverpool, Liverpool, United Kingdom; PLOS: Public Library of Science, UNITED STATES

## Abstract

Maternal adverse childhood experiences (ACEs) have significant impacts on the next generation with links to negative birth outcomes, impaired cognitive development, and increased socioemotional problems in children. However, not all types or levels of adversity are similarly deleterious and research from diverse contexts is needed to better understand why and how intergenerational transmission of adversity occurs. We examined the role of maternal ACEs on children’s growth, cognitive, and socioemotional development at 36 months postpartum in rural Pakistan. We used data from 877 mother-child dyads in the Bachpan Cohort, a birth cohort study. Maternal ACEs were captured using an adapted version of the ACE-International Questionnaire. Outcomes at 36 months of age included child growth using the WHO growth z-scores, fine motor and receptive language development assessed with the Bayley Scales of Infant and Toddler Development, and socioemotional and behavioral development measured with the Ages and Stages Questionnaire: Socioemotional and Strengths and Difficulties Questionnaire. To estimate the associations between maternal ACEs and child outcomes, we used multivariable generalized linear models with inverse probability weights to account for sampling and loss to follow-up. Over half of mothers in our sample (58%) experienced at least one ACE. Emotional abuse, physical abuse, and emotional neglect were the most commonly reported ACEs. We found null relationships between the number of maternal ACEs and child growth. Maternal ACEs were associated with higher fine motor and receptive language development and worse socioemotional and behavioral outcomes. Maternal ACE domains had similarly varying relationships with child outcomes. Our findings highlight the complexity of intergenerational associations between maternal ACEs and children’s growth and development. Further work is necessary to examine these relationships across cultural contexts and identify moderating factors to mitigate potential negative intergenerational effects.

## Introduction

Adverse childhood experiences (ACEs) refer to severely stressful exposures or experiences that occur in childhood, such as abuse, neglect, violence between caregivers, and peer or community violence [1]. The effects of ACEs start early in life and continue throughout the lifecourse, beginning with delayed child development and progressing to poor psychological and physical health outcomes in adulthood [2,3]. ACEs have been linked to numerous adult health outcomes such as cardiometabolic disease, cancer, mortality [4–6], and several negative psychological outcomes including depression, post-traumatic stress disorder, anxiety, and suicide [7–10]. Severe early life stressors can lead to observable changes in brain structure and function, which has potentially permanent effects on development [11,12], and can alter physiological systems, such as the stress response axes, immune functioning, and inflammation [13].

While there is considerable evidence of the harmful impact of accumulated ACEs across an individual’s life course, intergenerational effects are an understudied area of concern. The intergenerational transmission of maternal childhood adversity may occur through biological embedding during pregnancy as well as through maternal mental health and parenting-related pathways [13,14]. For example, stress-induced epigenetic alterations during a mother’s childhood can alter the maternal-placental-fetal endocrine, immune, and inflammatory stress biology. Such alterations may lead to biological changes across multiple systems and ultimately affect children’s physical, cognitive, and socioemotional development [15]. A recent study identified a link between maternal ACEs and newborn brain development, which was subsequently associated with negative infant emotionality [16]. Maternal ACEs may also impact the quality of the postnatal environment [14] and, indeed, maternal anxiety, depression, and parenting practices are key mediators between maternal ACEs and child socioemotional development [17–20].

In relation to parental ACEs and early childhood development, the most studied domain is socioemotional development. Worse socioemotional functioning, evident through greater externalizing and internalizing problems, has been shown to exist for children born to mothers with greater exposure to ACEs in high-income countries [17,21–23]. These socioemotional problems include higher anxiety, aggression, hyperactivity, and negative affect in the first three years of life [9,24,25].

Less research has been conducted examining the impact of maternal childhood adversity and the next generation’s physical growth and cognitive development. Examination of the relationship with children’s physical growth is important because linear growth in early childhood is linked to later cognitive and socioemotional development [26,27]. Of the existing research on intergenerational effects, maternal ACEs have been linked to low birth weight and shorter gestational age [28], lower overall development at 12 months [29], lower parental-rated physical health at 18 months [30], and decreased problem solving, gross motor, fine motor, and communication skills at 24 months [31]. All of these studies were conducted in the United States (US) or Canada, and few studies have examined the intergenerational effects of maternal ACE exposure in low- and middle- income countries (LMICs). Addressing ACEs in LMICs is critically important for several reasons: adversity in childhood is more prevalent in LMICs, resources to address ACEs in LMICs are limited, and the majority of the world’s population resides in LMICs [32]. Finally, understanding the intergenerational effects of maternal ACEs across cultural contexts will help to identify vulnerable populations for intervention.

To our knowledge, we are only aware of three studies assessing the intergenerational effects of maternal ACEs in LMICs. In one recent study, researchers reported that maternal ACEs were associated with poor fetal attachment during pregnancy across eight LMICs; however, the effects varied across cultural contexts [33]. Researchers reported positive effect estimates of maternal ACEs on fetal attachment in Jamaica, Pakistan, Philippines, and South Africa, no effect in Sri Lanka, and negative effect estimates in Ghana, Romania, and Vietnam. Two other studies found maternal ACEs were associated with worse socioemotional development among older children (aged 2–18 years) in Kenya [19,20]. The heterogeneity of results from these studies underscore the importance of studying the intergenerational effects of maternal childhood adversity across diverse contexts. These studies also did not investigate how maternal ACEs affected early child growth and development.

The present study examined the role of maternal ACEs in child growth and development at 36 months postpartum in rural Pakistan. Using data on 877 mother-child dyads from a birth cohort, we hypothesized children of mothers with greater exposure to ACEs would experience poorer growth, cognitive development, and worse socioemotional and behavioral outcomes.

## Methods

### Study population and participants

We used data from the Bachpan Cohort, a birth cohort study based in Kallar Syedan, a rural subdistrict in Rawalpindi, Punjab Province [34,35]. Kallar Syedan has a population of roughly 200,000 people and the average household size is six individuals [36]. Literacy rates for men and women in rural Punjab province are 70% and 53%, respectively. Women in our sample had higher educational attainment compared to the Demographic and Health Survey (DHS) rates in rural areas of the Punjab province (50% in our sample achieved secondary school or higher vs. 20% in the DHS) [37].

The Bachpan Cohort is a longitudinal study with an embedded perinatal depression intervention trial. Details on the cohort can be found elsewhere [34,35,38,39]. From 2014–2016, all pregnant women in their third trimester in 40 village clusters were screened for depression using the Patient Health Questionnaire (PHQ-9) [40]. Women who screened positive for depression (PHQ-9 ≥ 10) were invited to participate in the trial and randomized to control or intervention arms. A random sample of women who screened negative for depression (PHQ-9 < 10) were asked to participate in the cohort only portion, which created a population-representative sample. Mother-child dyads were enrolled in their third trimester and followed up at 3, 6, 12, 24, and 36 months postpartum. Participants were allowed to return to the study if the mother and child were still alive and living in the study area. Of the 1,154 women assessed at baseline, 265 were lost to follow-up by 36 months and 12 were missing outcome measurements, resulting in 877 mother-child dyads with complete data.

### Exposure

We measured maternal ACEs using an adapted version of the ACE-International Questionnaire (ACE-IQ) [41], a self-reported retrospective measure that has been validated in other low-resource contexts (S1 Table) [42,43]. The ACE-IQ was adapted from the original ACE measure developed in the US [44] to include items on peer violence, exposure to collective violence, and witnessing community violence. Due to potential risks to the participant and expected underreporting, we removed the sexual abuse questions. At 36 months postpartum, mothers retrospectively reported their experiences of 12 adverse exposures in childhood.

We operationalized maternal ACEs in multiple ways. We created a continuous score ranging from 0–12. We also generated a categorical variable with the number of experiences (one, two, three, and four or more). Additionally, we created domain-specific indicators for neglect (emotional and physical), household psychological distress (alcohol and/or drug abuser in the household; incarcerated household member; someone depressed, mentally ill, institutionalized or suicidal), home violence (physical abuse; emotional abuse; household member treated violently), and community violence (bullying; community violence; collective violence). For each domain-specific indicator, women received a “Yes” if they experienced any of the ACEs within each domain. Twenty women responded “Do not remember” to some ACE questions and these were recoded as “No” after sensitivity analyses coding these cases as missing did not qualitatively change the results.

### Outcomes

Our main outcomes were child growth, fine motor skills, receptive language development, and socioemotional and behavioral outcomes at 36 months. We used the World Health Organization’s international standards to measure physical growth z-scores using length-for-age (LAZ), weight-for-age (WAZ), and weight-for-length (WLZ) [45]. We measured fine motor and receptive language development using the Bayley Scales of Infant and Toddler Development (BSITD), third edition [46]. Scaled scores were calculated using a reference population in the US by the child’s age group; scores range from 1–19 with a mean of 10 and standard deviation of three. The BSITD has been widely used internationally and validated in similar contexts to our study setting [47,48]. Socioemotional development was captured using the Ages and Stages Questionnaire: Socioemotional (ASQ:SE) [49]. The ASQ:SE is a 30-item parent-reported measure, theoretically ranging from 0–270, with higher scores indicating worse child socioemotional development. Behavioral outcomes were assessed using the parent-reported Strengths and Difficulties Questionnaire (SDQ) [50]. The SDQ covers 25 questions in five domains: hyperactivity, emotional, conduct, and peer problems, and pro-social behaviors. A total score is calculated by summing the first four domains and theoretically ranges from 0–40, with higher scores indicating worse behavioral outcomes. The ASQ:SE and SDQ are commonly used in LMICs and have been found to be reliable and valid across cultural settings [51,52].

### Confounders

We included the following baseline confounders informed by a directed acyclic graph (DAG) [53,54] to estimate the total effect of maternal ACEs on early child growth and development: maternal age, education, and family history of mental illness. We aimed to estimate the total effect of maternal ACEs on child outcomes; therefore, we did not control for potential variables, such as maternal depression or child health, which we identified as mediators using the DAG (S1 Fig). We used maternal education as a proxy for maternal childhood socioeconomic status and operationalized it as a categorical variable (none, primary or middle school, secondary education or higher). We asked mothers to report if anyone in her immediate natal family had an existing mental illness and used it as a proxy for the mother’s family history of mental illness.

### Statistical analysis

We constructed multivariable generalized linear models to assess the relationship between maternal ACEs and child growth and development. In addition to confounders, we also included child gender, trial arm, and assessor as auxiliary variables in all models to improve precision [55]. We used cluster robust standard errors to account for clustering by village, and cluster-specific sampling weights to account for unequal sampling probabilities by baseline depression. In a given cluster, all non-depressed women were weighted by the inverse of their sampling fraction (i.e., the proportion that were screened for depression at recruitment and subsequently enrolled), and depressed women were each given a weight of 1. Therefore, non-depressed women were upweighted to account for their lower probability of selection into the study.

To account for informative censoring between baseline and 36 months postpartum, we used stabilized inverse probability of censoring weights (IPCW) [56]. IPCW are estimated as the inverse of the probability that a woman was not censored at 36 months, based on observed characteristics. Women who were not loss to follow-up were upweighted to represent those who were lost to follow-up. In the IPCW model, we included baseline confounders (maternal age, education, and family history of mental illness) and baseline characteristics associated with censoring using a p-value <0.10 (maternal depression, child’s grandmother co-residence, number of people per room, and nuclear vs. joint family), with household asset scores included to increase precision [57]. IPCW were stabilized by using the marginal probability of being observed in the numerator. Sampling weights and IPCW were multiplied to create a final weight used in all models [58]. The final weight was also used to estimate means and frequencies in descriptive tables; numbers were unweighted. Analyses were conducted in Stata 14 and R (4.1.0).

### Ethics approval

The Bachpan Cohort study received ethical approval from the institutional review boards at the Human Development Research Foundation (IRB/1017/2021), Duke University, and the University of North Carolina at Chapel Hill (#20–1433). Written informed consent, or witnessed informed consent if the participant was illiterate, was obtained before study participation.

## Results

### Descriptive statistics

There were 877 women and children in our analytic sample (Table 1). After applying weights, on average, women were 27 years old and half had completed secondary school education or higher at baseline. Roughly 28% of mothers were diagnosed with depression at baseline and 9% reported having a family history of mental illness. The majority of households were joint or extended families and 68% of children were co-residing with a grandmother. Almost half of the children were female and mean LAZ and WAZ were -0.99 (SD = 1.10) and -0.96 (SD = 0.99), respectively. Mean fine motor and receptive language scaled scores were slightly higher than the average of 10 (10.43 [2.79]; 11.45 [4.03], respectively). Mean ASQ-SE and SDQ scores were 38.83 (SD = 17.03) and 14.02 (SD = 6.30), respectively.

**Table 1:** Sample characteristics, Bachpan Cohort, Pakistan, n = 877.

		Mean or N	SD or %	Range
**Maternal characteristics (baseline)**				
Maternal age		26.60	4.29	18–45
Maternal education				
	None	124	13.60	
	Primary or middle school	338	36.27	
	Secondary school or higher	413	50.14	
Natal family history of mental illness		95	8.93	
Depression diagnosis (SCID)		314	27.56	
**Household characteristics (baseline)**				
People per room		2.37	1.87	0–23
Nuclear family (vs. joint or extended)		111	13.16	
Grandmother coresidence		609	68.29	
Trial arm: Intervention (vs. control)		426	51.68	
Household assets*				
	Lowest	165	17.58	
	Lower middle	176	18.37	
	Middle	184	19.68	
	Upper middle	173	21.99	
	Highest	177	22.38	
**Child characteristics (36 months)**				
	Age (months)	36.3	0.60	
	Gender: Female	432	48.37	
	Length-for-age z-score	-0.99	1.10	-4.59–2.87
	Weight-for-age z-score	-0.96	0.99	-4.64–4.23
	Weight-for-height z-score	-0.61	1.12	-5.08–5.65
	BSITD receptive language scaled score	10.43	2.79	5–19
	BSITD fine motor scaled score	11.45	4.03	2–19
	ASQ-SE total score	38.83	17.03	10–170
	SDQ total difficulties score	14.02	6.30	3–34

* Sampling probability and inverse probability weights were used to calculate means, SDs, and percentages.

Abbreviations: Bayley Scales of Infant and Toddler Development (BSITD); Ages and Stages Questionnaire-Socioemotional (ASQ-SE); Strengths and Difficulties Questionnaire (SDQ)

* Principal components analysis was used to create an asset index and then operationalized as quintiles.

After applying weights, over 58% of women (n = 512) experienced at least one ACE (Table 2). Among the 12 ACE questions, emotional abuse (n = 290, 32%), physical abuse (n = 206, 23%), emotional neglect (n = 131, 15%), and witnessing a household member being treated violently (n = 128, 15%) were the most common. With respect to ACE domains, more than one in three women were exposed to violence in their childhood home (n = 344, 39%) and one in five experienced emotional or physical neglect during their childhood (n = 170, 19%). Roughly 16% (n = 141) experienced family psychological distress. Community violence was far less common (n = 63, 6.9%).

**Table 2:** Maternal Adverse Childhood Experiences (n = 877)*.

		N	%
**Number of ACEs**			
	None	363	41.49
	1	235	26.86
	2	137	15.66
	3	81	9.26
	4 or more	59	6.74
**Neglect**			
	Physical neglect	49	5.60
	Emotional neglect	131	14.97
**Family psychological distress**			
	One or no parents, parental separation or divorce	96	10.97
	Alcohol/drug abuser in the household	23	2.63
	Someone chronically depressed, mentally ill	21	2.40
	Incarcerated household member	16	1.83
**Home violence**			
	Household member treated violently	128	14.63
	Physical abuse	206	23.54
	Emotional abuse	290	33.14
**Community violence**			
	Bullying	12	1.37
	Community violence	59	6.74
	Collective violence	4	0.46
**ACE domains** **			
	Neglect	170	19.43
	Family psychological distress	141	16.11
	Home violence	344	39.31
	Community violence	63	7.20
		**Mean**	**SD**
**Total number of ACEs** (range: 0–10)		2.37	1.38

* Sampling probability weights and inverse probability weights were used.

** Domains were binary categories describing experience of any individual ACEs.

We highlight the effects of maternal ACEs on child growth and development below. Full estimates and precision are presented in S2 and S3 Tables.

### Growth outcomes

**Fig 1:**
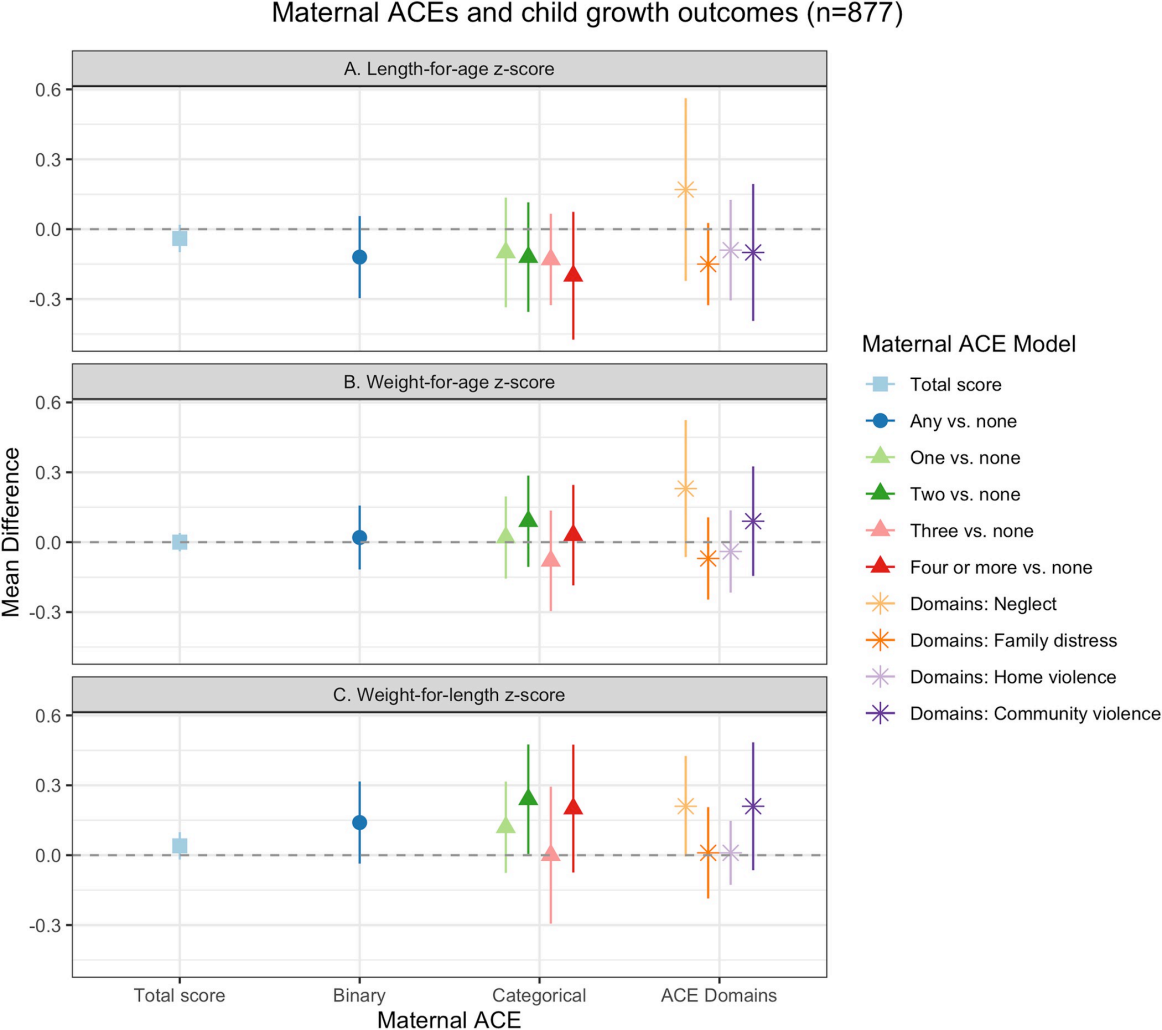
Maternal ACEs and child growth, Bachpan Cohort, Pakistan (n = 877). We used weighted generalized linear models with cluster robust standard errors. Sampling and inverse probability censoring weights were combined. All models controlled for baseline maternal age, maternal education, trial arm, assessor, and child gender.

We found cross-sectional associations between maternal ACEs and child growth z-scores (Fig 1, S2 Table). There were small, negative relationships between maternal ACEs and child LAZ (Fig 1A). We found a small, negative stepwise trend between categorical maternal ACEs and child LAZ. Compared to no maternal ACEs, the mean difference of having an additional maternal ACE on child LAZ became stronger as the number of ACEs increased, with one maternal ACE associated with a 0.10 standard deviation (SD) decrease in child LAZ (95% CI: -0.34, 0.14) and four or more maternal ACEs associated with a 0.20 SD decrease in child LAZ (95% CI: -0.48, 0.09). Maternal childhood exposure to neglect was associated with a 0.17 SD increase in child LAZ (95% CI: -0.23, 0.56), controlling for other ACE domains. Family distress and community violence had small, negative associations with child LAZ (Fig 1A) (Family distress: MD = -0.15 (95% CI: -0.32, 0.03); Community violence: MD = -0.10 (95% CI: -0.40, 0.21).

We found largely null relationships between maternal ACEs and child WAZ (Fig 1B); however, neglect was associated with a 0.23 SD increase in WAZ, controlling for other ACE domains (95% CI: -0.07, 0.53). Maternal ACEs and child WLZ had small positive associations (Fig 1C). Any maternal ACE compared to none was associated with a 0.14 SD increase in WLZ (95% CI: -0.03, 0.31). There was no clear stepwise trend between categorical maternal ACEs and WLZ; however, four or more maternal ACEs were associated with a 0.20 SD higher WLZ (95% CI: -0.08, 0.48). Maternal childhood exposure to neglect and community violence were also each associated with a 0.21 increase in child WLZ (95% CI: -0.02, 0.44; 95% CI: -0.07, 0.50, respectively).

### Fine motor and receptive language outcomes

**Fig 2:**
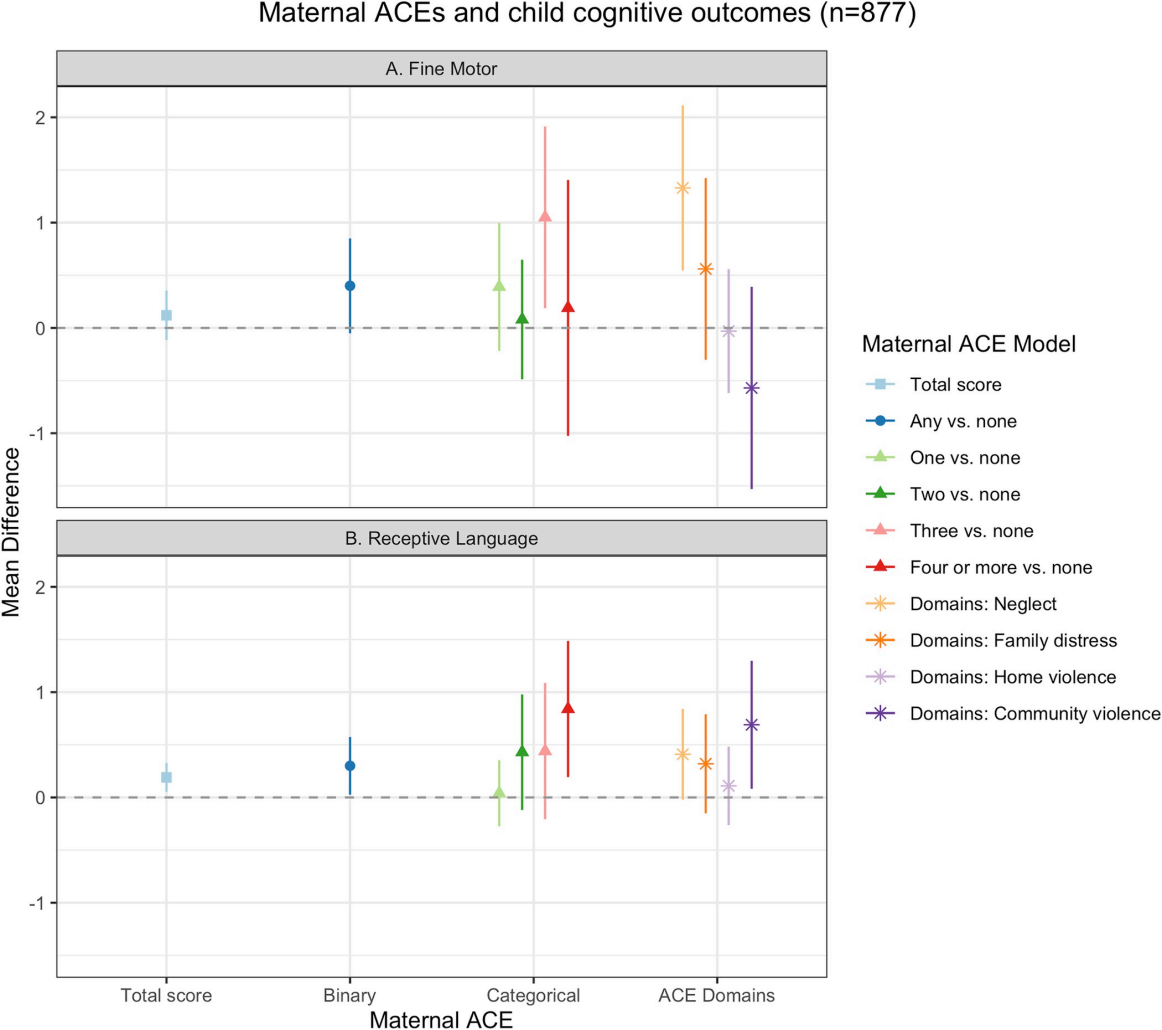
Maternal ACEs and child fine motor and receptive language development, Bachpan Cohort, Pakistan (n = 877). We used weighted generalized linear models with cluster robust standard errors. Sampling and inverse probability censoring weights were combined. All models controlled for baseline maternal age, maternal education, trial arm, assessor, and child gender.

We found positive associations between maternal ACEs and children’s fine motor and receptive language scores (Fig 2, S3 Table). Any maternal experience of ACEs was associated with a 0.40-point increase in child fine motor scores (95% CI: -0.07, 0.87) compared to no maternal ACE. There was no clear stepwise trend in categorical maternal ACE exposure and fine motor scores (Fig 2A); however, we found small positive associations between mothers experiencing one ACE (versus none) and three ACEs (versus none) and child fine motor scores (One maternal ACE: MD = 0.39 (95% CI: -0.22, 1.01); Three maternal ACEs: MD = 1.05 (95% CI: 0.15, 1.94)). Maternal childhood experiences of neglect and family distress were associated with higher child fine motor scores (Neglect: MD = 1.33 (95% CI: 0.52, 2.14); Family distress: MD = 0.56 (95% CI: -0.32, 1.44)) (Fig 2A). Community violence was associated with lower fine motor scores (MD = -0.57 (95% CI: -1.56, 0.42).

Similar to the fine motor results, we found positive associations between maternal ACE and child receptive language skills. Any maternal ACE was associated with a 0.30-point increase in receptive language scores (95% CI: 0.02, 0.59), compared to no maternal ACE. We saw a small stepwise trend between categorical maternal ACEs and child receptive language scores; there was no association with one maternal ACE, but an increasingly small positive association with two, three, and four or more ACEs, with four or more maternal ACEs associated with a 0.84-point increase in scores (95% CI: 0.16, 1.51) (Fig 2B). Controlling for the other ACE domains, we found positive, but small independent associations between neglect, family distress, and home violence and receptive language scores (Neglect: MD = 0.41 (95% CI: -0.04, 0.86); Family distress: MD = 0.32 (95% CI: -0.17, 0.82); Community violence: MD = -0.73 (95% CI: 0.09, 1.38)) (Fig 2B). Home violence was not associated with children’s fine motor or receptive language scores.

### Socioemotional outcomes

**Fig 3:**
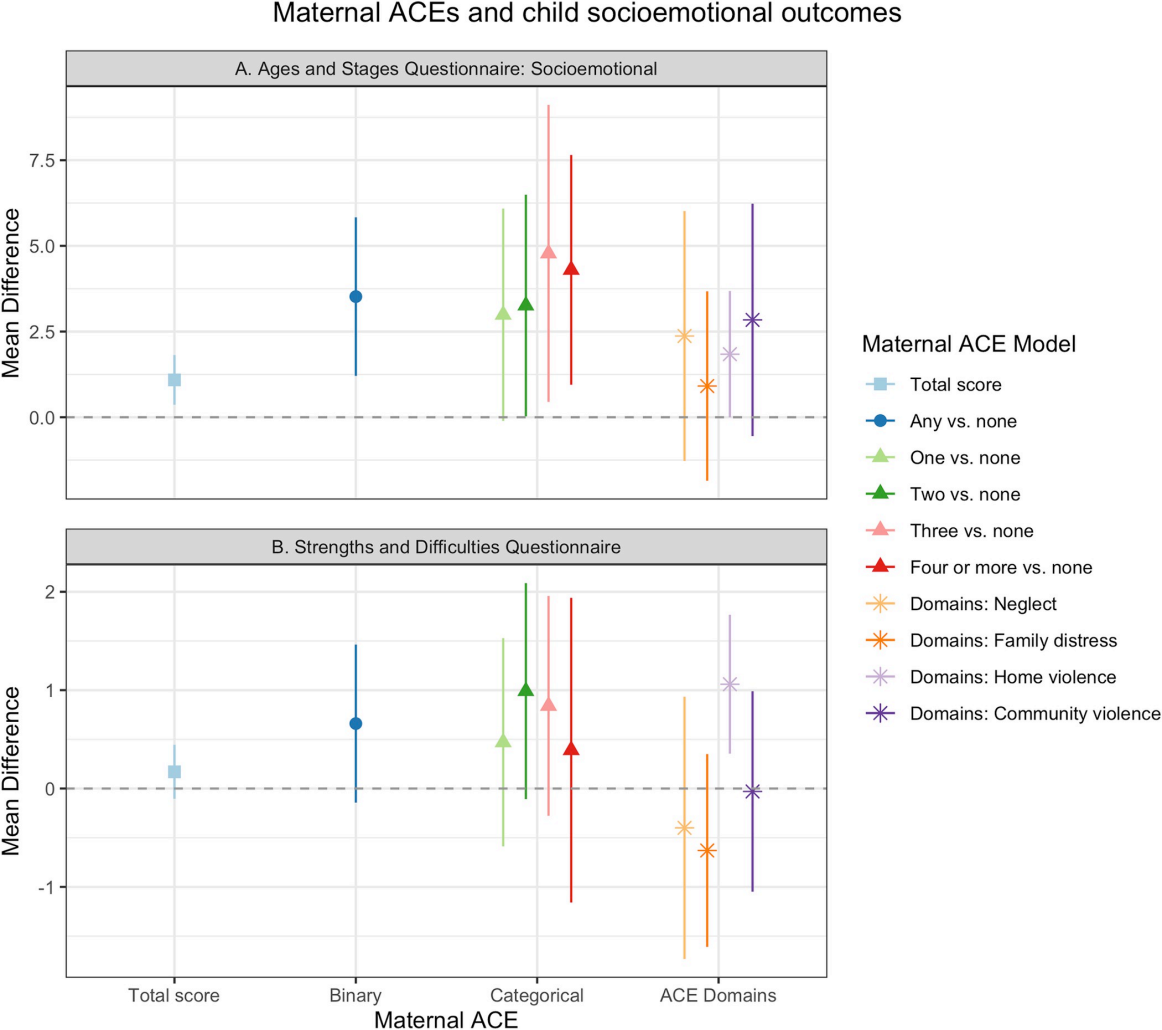
Maternal ACEs and child socioemotional and behavioral outcomes, Bachpan Cohort, Pakistan (n = 877). We used weighted generalized linear models with cluster robust standard errors. Sampling and inverse probability censoring weights were combined. All models controlled for baseline maternal age, maternal education, trial arm, assessor, and child gender.

We found that maternal ACEs were associated with higher ASQ:SE and SDQ scores for children, which suggests worse child socioemotional and behavioral development (Fig 3, S3 Table). Compared to children of mothers who experienced no ACE, children whose mothers experienced any ACE scored 3.52 points higher on the ASQ:SE (Fig 3A, 95% CI: 1.14, 5.90). Categorical maternal ACEs (one, two, three, and four or more) were associated with increasingly higher ASQ:SE scores compared to no maternal ACE (Fig 3A). One maternal ACE exposure was associated with a 2.99-point increase in ASQ:SE (95% CI: -0.21, 6.18) and four or more ACEs associated with a 4.30-point increase (95% CI: 0.84, 7.75). Maternal experience of all four domains (neglect, family distress, home violence, and community violence) were associated with roughly 1–3 points higher ASQ:SE scores (Fig 3A); however, our estimates were imprecise as indicated by the wide confidence intervals with confidence limit differences ranging from 3.79 to 7.53.

We found a similar effect of maternal ACEs on SDQ; however, the results were attenuated compared to ASQ:SE. Any maternal ACE was associated with a 0.66-point increase in SDQ scores compared to no ACE (Fig 3B, 95% CI: -0.17, 1.50). We found a small increasing pattern between categorical maternal ACEs and SDQ scores (Fig 3B). Maternal childhood experience of home violence was associated with 1.06-point increase in children’s SDQ scores (Fig 3B, (95% CI: 0.32, 1.80), while neglect and family distress had small negative associations with child SDQ scores (Neglect: MD = -0.40 (95% CI: -1.77, 0.97); Family distress: MD = -0.63 (95% CI: -1.64, 0.39)).

## Discussion

The goal of our study was to estimate the relationships between maternal ACEs and child development at 36 months of age. Over half of the mothers in our sample in rural Pakistan experienced at least one ACE. Maternal ACEs were not strongly associated with child growth z-scores. We found an unexpected relationship between maternal ACEs and child fine motor and receptive language development, where maternal childhood experience of adversity was associated with more positive child development. Maternal ACEs were associated with worse child socioemotional and behavioral outcomes.

Mothers most commonly reported experiences of emotional abuse, physical abuse, emotional neglect, or seeing a household member treated violently during their childhood. The prevalence of any maternal ACEs in our sample (58%) was lower than in other LMICs using the same measure (ACE-IQ, roughly 80% in Saudi Arabia, Vietnam, Tunisia, and Lebanon) [59]. Other studies in Pakistan have also reported lower levels of ACEs. For example, one study of Pakistani university students found the mean number of ACEs among women was 0.40 (SD = 0.92) [60], compared with our mean of 2.37 (SD = 1.38). The authors of that study emphasized that in a collectivist and conservative context like Pakistan, the role of the family is critical even throughout adulthood, making it especially difficult to disclose sensitive topics, such as child maltreatment and family distress. Another study of pregnant women in Pakistan reported a similar mean number of maternal ACEs in Pakistan (3.07, SD = 2.37) as our study (2.37, SD = 1.38) [33]. This study also found a wide range of mean ACEs across eight LMICs (from 2.54 in Vietnam to 6.42 in South Africa) [33], thus underscoring the importance of examining diverse cultural and contextual factors when assessing ACEs. Specific to measurement, another reason for the low prevalence of ACEs in our study may be due to our exclusion of the sexual abuse questions, given potential risks to participants and expected underreporting of disclosure. Cross-country variation in the mean number of reported ACEs may therefore be due to both differential reporting and true variation in ACE exposures.

In our study, maternal ACEs were not strongly associated with child growth outcomes at 36 months. If a key mechanism through which maternal ACEs influence growth is through the intrauterine environment in pregnancy [13,14], it is possible that any such effects would be observed closer to birth. The potential negative effects of maternal ACEs on children’s physical development could have also been remedied through moderating factors, such as improved nutrition and catch-up growth [61]. During the first three years of life, other external factors might also have stronger influences on child growth. In low-resource contexts, other forms of early life adversity not captured by ACEs, such as extreme poverty, may have a greater effect on children’s physical development than maternal ACEs [62]. Future studies examining these potential mediators between maternal ACEs and child growth would help identify possible intergenerational pathways.

Notably, maternal ACEs were associated with higher children’s fine motor and receptive language scores. There is limited literature examining the intergenerational transmission of maternal ACEs on children’s overall developmental outcomes. Existing research from high-income countries are mixed. In two studies from the US and Canada, maternal ACEs were associated with worse overall development and greater risk of developmental delay [29,31], while another US study found no relationship between maternal ACEs and child development [63]. We found no prior study investigating maternal ACEs and overall child development in LMICs. While more research is needed to replicate these findings, life history evolution theory may help to explain the positive relationship between maternal ACEs and better child development. Life history theory suggests that individuals who are exposed to harsh or unpredictable environments (e.g. poverty, exposure to violence, harsh parenting) develop faster life history strategies in order to adapt and survive in such stressful environments [64,65]. Existing evidence demonstrates that parents with ACEs were less emotionally available and engaged in harsh discipline with their children [66]. While outside the scope of this study, future studies should examine the relationship between maternal ACEs and parenting behaviors, such as maternal responsivity and quality of the home environment.

Related, post-traumatic growth may also help explain a positive relationship between maternal ACEs and better child development. Post-traumatic growth is defined as a positive psychological change following traumatic events [67–69]. Prior work has found that individuals who experience past adversity have increased empathy and altruism for others [70–72]. In support of this hypothesis, Brown et al (2021) found a positive effect of maternal ACEs on fetal attachment among Pakistani mothers, although they did not examine child development. Importantly, the exact mechanisms through which this would be linked to improved developmental outcomes remains unknown. Assessing the ways in which post-traumatic growth among mothers who experienced ACEs affects health and social behaviors across the lifecourse would be invaluable.

In contrast to the findings with overall development, maternal ACEs were associated with worse child socioemotional development and behavioral outcomes in our sample. This finding is consistent with the existing literature. In a systematic review, authors found maternal ACEs were associated with child externalizing behavior problems and the majority of included studies found relationships with child internalizing problems [73]. Similar results have been found in Kenya among older aged children (2–18 years) [19,20]. These links can potentially be explained through both biological and psychological pathways. Potential biological mechanisms suggest maternal experience of childhood adversity may influence maternal HPA-axis functioning during pregnancy, leading to changes in the gestational environment and the development of fetal stress response systems [74,75]. The hormonal changes during pregnancy may, in turn, influence the woman’s mental health and health behaviors [76,77], which can then affect infant stress regulation and behavioral difficulties [78]. Psychosocial mechanisms posit that maternal ACEs can lead to poor attachment and development of healthy relationships between mothers and children [73]. Mothers who experience childhood trauma may be predisposed to mental health conditions such as anxiety or depression, and poor maternal mental health has been linked to increased parenting stress, impaired mother-child interactions, and harsher maternal parenting practices [79]. Furthermore, in the context of LMICs, economic and parenting resources may be scarce, which can exacerbate stress in the home environment. This, in turn, may lead to suboptimal caregiving (lack of warmth and responsiveness) and ultimately, worse child socioemotional development and behavior outcomes.

Finally, maternal ACE domains (Neglect, Family distress, Home violence, and Community violence) had varying relationships with child development. Maternal childhood exposure to neglect was associated with better child growth, fine motor, and receptive language development, but worse child socioemotional development. Maternal childhood experience of family distress, home violence, and community violence had varied relationships with child outcomes. It is important to note that deprivation (absence of cognitive and social inputs) and threat (presence of threatening experiences) likely have distinct influences on neural development [80], and research has shown differential impacts on cognitive and emotional outcomes in children as well as pregnant women [81–83]. As previously mentioned, post-traumatic growth and compensatory parenting practices may help to explain the positive relationships of past maternal neglect on her own child’s outcomes [67,68,84]. Positive experiences, such as supportive relationships and prosocial activities, can help to mitigate the impact of ACEs by serving as a promotive resilience factor [84]. Prior research demonstrates that adults who had positive childhood experiences are more likely to have resilient functioning and more compensatory behaviors (e.g. less harsh parenting attitudes, greater affection) even if they experienced ACEs [85–89]. Mothers who experienced neglect in their childhood and had positive experiences concurrently or later in childhood may develop resiliency and in turn provide more resources and improved parenting for their children. Future research in LMICs should focus on identifying differential impacts of ACE domains on the next generation and examine whether and how positive experiences can mitigate the intergenerational transmission.

Although such explorations are beyond the scope of the current paper, important factors, such as maternal depression and parenting practices, may mediate the relationship between maternal ACEs and child outcomes. Previous work from our group demonstrated that ACEs were associated with greater depressive symptoms and major depressive episodes [90]. Studies in Kenya found that maternal mental health mediated the relationship between maternal ACEs and child mental health problems [19,20]. A study among pregnant mothers across eight LMICs found prenatal depression fully mediated the effect of maternal ACEs on fetal attachment using the full sample [33]. However, at the country-level, direct effects varied; in Vietnam, maternal ACEs directly and negatively affected fetal attachment and maternal prenatal depression did not mediate the relationship [33]. Other caregivers, such as fathers and grandmothers, are also important to consider. Their contributions may buffer any negative effects of maternal ACEs on children by improving maternal social support.

### Strengths and limitations

Our study had several strengths. We used standard, validated measures of child outcomes that have been previously used in our study context. Moreover, we estimated the total effect of maternal ACEs on the next generation’s development as opposed to controlling for potential mediators, such as adult socioeconomic status or depression status, which may lead to biased estimates. Our study also had limitations that require discussion. First, although we used the ACE-IQ to capture maternal ACEs, it is a retrospective measure, which is vulnerable to recall bias. In the context of this study, it may be that depressed women are more likely to report ACEs in order to understand their depressive symptoms, resulting in potential differential misclassification. Moreover, the ACE-IQ may not fully measure adversities during childhood in this context. Second, while we controlled for maternal childhood SES and maternal family history of mental illness, we used proxies (maternal education and natal family mental illness history, respectively), and residual confounding is possible. Third, 265 women and children were lost to follow-up at 36 months postpartum; however, we used stabilized IPCW to account for informative censoring. Finally, there may be unmeasured confounders that affect this relationship, such as how mothers were raised (i.e., the caregiving practices of the mother’s parents).

## Conclusion

Our findings suggest the intergenerational transmission of childhood adversity is complex and may differentially influence physical, cognitive, and socioemotional domains of child development. To improve child health and development, it is necessary to understand maternal life histories, including her childhood experiences, rather than only capturing her experiences during pregnancy and postpartum. Future work should examine the intergenerational relationship of childhood adversity across cultural contexts in order to understand specific risk and protective factors and develop tailored interventions. Identifying moderating factors, such as promoting maternal responsive caregiving and improving maternal social support via other caregivers, can help inform strategies to disrupt the intergenerational cycle of adversity that is responsive to traumatic histories.

## Supporting information

S1 Fig Maternal ACEs and child development directed acyclic graph. (DOCX)

S1 Table Adapted Adverse Childhood Experiences International Questionnaire (ACEs-IQ). (DOCX)

S2 Table Maternal ACEs and child growth, Bachpan Cohort, Pakistan (n = 877). (DOCX)

S3 Table Maternal ACEs and child development, Bachpan Cohort, Pakistan (n = 877). (DOCX)

S1 Dataset Bachpan dataset. (DTA)

## References

[1] Centers for Disease Control and Prevention. Preventing Adverse Childhood Experiences. Atlanta, GA: National Center for Injury Prevention and Control, Centers for Disease Control and Prevention; 2019.

[2] Cprek SE, Williamson LH, McDaniel H, Brase R, Williams CM. Adverse Childhood Experiences (ACEs) and Risk of Childhood Delays in Children Ages 1–5. Child Adolesc Soc Work J. 2020;37(1):15–24.

[3] Shonkoff JP, Garner AS, Committee on Psychosocial Aspects of C, Family H, Committee on Early Childhood A, Dependent C, et al. The lifelong effects of early childhood adversity and toxic stress. Pediatrics. 2012 Jan;129(1):e232–46. 22201156 10.1542/peds.2011-2663

[4] Brown DW, Anda RF, Tiemeier H, Felitti VJ, Edwards VJ, Croft JB, et al. Adverse childhood experiences and the risk of premature mortality. Am J Prev Med. 2009 Nov;37(5):389–96. doi: 10.1016/j.amepre.2009.06.021 19840693

[5] Brown MJ, Thacker LR, Cohen SA. Association between adverse childhood experiences and diagnosis of cancer. PloS One. 2013;8(6):e65524. doi: 10.1371/journal.pone.0065524 23776494 PMC3679131

[6] Jakubowski KP, Cundiff JM, Matthews KA. Cumulative childhood adversity and adult cardiometabolic disease: A meta-analysis. Health Psychol Off J Div Health Psychol Am Psychol Assoc. 2018 Aug;37(8):701–15. doi: 10.1037/hea0000637 30024227 PMC6109976

[7] Atzl VM, Narayan AJ, Rivera LM, Lieberman AF. Adverse childhood experiences and prenatal mental health: Type of ACEs and age of maltreatment onset. J Fam Psychol JFP J Div Fam Psychol Am Psychol Assoc Div 43. 2019 Apr;33(3):304–14. doi: 10.1037/fam0000510 30802085

[8] Chapman DP, Whitfield CL, Felitti VJ, Dube SR, Edwards VJ, Anda RF. Adverse childhood experiences and the risk of depressive disorders in adulthood. J Affect Disord. 2004 Oct 15;82(2):217–25. doi: 10.1016/j.jad.2003.12.013 15488250

[9] Letourneau N, Dewey D, Kaplan BJ, Ntanda H, Novick J, Thomas JC, et al. Intergenerational transmission of adverse childhood experiences via maternal depression and anxiety and moderation by child sex. J Dev Orig Health Dis. 2019 Feb;10(1):88–99. doi: 10.1017/S2040174418000648 30175696

[10] Pournaghash-Tehrani SS, Zamanian H, Amini-Tehrani M. The Impact of Relational Adverse Childhood Experiences on Suicide Outcomes During Early and Young Adulthood. J Interpers Violence. 2019 May 29;886260519852160. doi: 10.1177/0886260519852160 31142213

[11] Juster RP, McEwen BS, Lupien SJ. Allostatic load biomarkers of chronic stress and impact on health and cognition. Neurosci Biobehav Rev. 2010;35(1):2–16. doi: 10.1016/j.neubiorev.2009.10.002 19822172

[12] McEwen BS, Gianaros PJ. Central role of the brain in stress and adaptation: links to socioeconomic status, health, and disease. Ann N Acad Sci. 2010;1186:190. doi: 10.1111/j.1749-6632.2009.05331.x 20201874 PMC2864527

[13] Berens AE, Jensen SKG, Nelson CA. Biological embedding of childhood adversity: from physiological mechanisms to clinical implications. BMC Med. 2017 Jul 20;15(1):135. doi: 10.1186/s12916-017-0895-4 28724431 PMC5518144

[14] Buss C, Entringer S, Moog NK, Toepfer P, Fair DA, Simhan HN, et al. Intergenerational Transmission of Maternal Childhood Maltreatment Exposure: Implications for Fetal Brain Development. J Am Acad Child Adolesc Psychiatry. 2017 May;56(5):373–82. doi: 10.1016/j.jaac.2017.03.001 28433086 PMC5402756

[15] Nelson CA, Bhutta ZA, Harris NB, Danese A, Samara M. Adversity in childhood is linked to mental and physical health throughout life. BMJ. 2020 Oct 28;371:m3048. doi: 10.1136/bmj.m3048 33115717 PMC7592151

[16] Demers CH, Hankin BL, Hennessey EMP, Haase MH, Bagonis MM, Kim SH, et al. Maternal adverse childhood experiences and infant subcortical brain volume. Neurobiol Stress. 2022 Nov 1;21:100487. doi: 10.1016/j.ynstr.2022.100487 36532374 PMC9755027

[17] Cooke JE, Racine N, Plamondon A, Tough S, Madigan S. Maternal adverse childhood experiences, attachment style, and mental health: Pathways of transmission to child behavior problems. Child Abuse Negl. 2019 Jul;93:27–37. doi: 10.1016/j.chiabu.2019.04.011 31048134

[18] Shih EW, Ahmad SI, Bush NR, Roubinov D, Tylavsky F, Graff C, et al. A path model examination: maternal anxiety and parenting mediate the association between maternal adverse childhood experiences and children’s internalizing behaviors. Psychol Med. 2021 May 18;1–11. doi: 10.1017/S0033291721001203 34001294 PMC9290334

[19] Kumar M, Amugune B, Madeghe B, Wambua GN, Osok J, Polkonikova-Wamoto A, et al. Mechanisms associated with maternal adverse childhood experiences on offspring’s mental health in Nairobi informal settlements: a mediational model testing approach. BMC Psychiatry. 2018 Dec;18(1):1–10. 30518351 10.1186/s12888-018-1953-y PMC6280351

[20] Rieder AD, Roth SL, Musyimi C, Ndetei D, Sassi RB, Mutiso V, et al. Impact of maternal adverse childhood experiences on child socioemotional function in rural Kenya: Mediating role of maternal mental health. Dev Sci. 2019 Sep;22(5):e12833. doi: 10.1111/desc.12833 30943319

[21] McDonnell CG, Valentino K. Intergenerational Effects of Childhood Trauma: Evaluating Pathways Among Maternal ACEs, Perinatal Depressive Symptoms, and Infant Outcomes. Child Maltreat. 2016 Nov;21(4):317–26. doi: 10.1177/1077559516659556 27457410

[22] Hetherington E, McDonal S, Tough S. Maternal Adverse Childhood Experiences (ACEs) and child development at 5 years. Paediatr Child Health. 2019;24(Supplement_2):e36–e36.

[23] Schickedanz A, Halfon N, Sastry N, Chung PJ. Parents’ adverse childhood experiences and their children’s behavioral health problems. Pediatrics. 2018;142(2):e20180023. doi: 10.1542/peds.2018-0023 29987168 PMC6317990

[24] McDonald SW, Madigan S, Racine N, Benzies K, Tomfohr L, Tough S. Maternal adverse childhood experiences, mental health, and child behaviour at age 3: The all our families community cohort study. Prev Med. 2019 Jan;118:286–94. doi: 10.1016/j.ypmed.2018.11.013 30468793

[25] Treat AE, Sheffield-Morris A, Williamson AC, Hays-Grudo J. Adverse childhood experiences and young children’s social and emotional development: the role of maternal depression, self-efficacy, and social support. Early Child Dev Care [Internet]. 2019 Jan 1 [cited 2020 Dec 16]; Available from: https://scholars.okstate.edu/en/publications/adverse-childhood-experiences-and-young-childrens-social-and-emot

[26] Sudfeld CR, Charles McCoy D, Danaei G, Fink G, Ezzati M, Andrews KG, et al. Linear Growth and Child Development in Low- and Middle-Income Countries: A Meta-Analysis. Pediatrics. 2015;135(5):e1266. doi: 10.1542/peds.2014-3111 25847806

[27] Prado EL, Larson LM, Cox K, Bettencourt K, Kubes JN, Shankar AH. Do effects of early life interventions on linear growth correspond to effects on neurobehavioural development? A systematic review and meta-analysis. Lancet Glob Health. 2019 Oct 1;7(10):e1398–413. doi: 10.1016/S2214-109X(19)30361-4 31537370

[28] Smith MV, Gotman N, Yonkers KA. Early Childhood Adversity and Pregnancy Outcomes. Matern Child Health J. 2016 Apr;20(4):790–8. doi: 10.1007/s10995-015-1909-5 26762511 PMC4849279

[29] Racine N, Plamondon A, Madigan S, McDonald S, Tough S. Maternal Adverse Childhood Experiences and Infant Development. Pediatrics. 2018;141(4):e20172495. doi: 10.1542/peds.2017-2495 29559588

[30] Madigan S, Wade M, Plamondon A, Maguire JL, Jenkins JM. Maternal Adverse Childhood Experience and Infant Health: Biomedical and Psychosocial Risks as Intermediary Mechanisms. J Pediatr. 2017 Aug;187:282–289.e1. doi: 10.1016/j.jpeds.2017.04.052 28549634

[31] Folger AT, Eismann EA, Stephenson NB, Shapiro RA, Macaluso M, Brownrigg ME, et al. Parental Adverse Childhood Experiences and Offspring Development at 2 Years of Age. Pediatrics. 2018;141(4):e20172826. doi: 10.1542/peds.2017-2826 29563236

[32] Mikton C, Butchart A. Current state of the global public health response to child maltreatment and intimate partner violence. In 2013.

[33] Brown RH, Eisner M, Walker S, Tomlinson M, Fearon P, Dunne MP, et al. The impact of maternal adverse childhood experiences and prenatal depressive symptoms on foetal attachment: Preliminary evidence from expectant mothers across eight middle-income countries. J Affect Disord. 2021 Dec 1;295:612–9. doi: 10.1016/j.jad.2021.08.066 34509077

[34] Turner EL, Sikander S, Bangash O, Zaidi A, Bates L, Gallis J, et al. The effectiveness of the peer-delivered Thinking Healthy PLUS (THPP+) Program for maternal depression and child socioemotional development in Pakistan: study protocol for a randomized controlled trial. Trials. 2016 Sep 8;17(1):442. 27608926 10.1186/s13063-016-1530-y PMC5017048

[35] Maselko J, Sikander S, Turner E, Bates L, Ahmad I, Atif N, et al. Effectiveness of a peer-delivered, psychosocial intervention on maternal depression and child development at 3 years postnatal: a cluster randomised trial in Pakistan. Lancet Psychiatry. 2020 Sep 1;7:775–87. doi: 10.1016/S2215-0366(20)30258-3 32828167 PMC8015797

[36] Government of Pakistan. Pakistan Bureau of Statistics Census. 2017.

[37] National Institute of Population Studies, ICF. Pakistan Demographic and Health Survey 2017–18 [Internet]. Islamabad, Pakistan: NIPS/Pakistan and ICF; 2019. Available from: http://dhsprogram.com/pubs/pdf/FR354/FR354.pdf.

[38] Sikander S, Ahmad I, Bates LM, Gallis J, Hagaman A, O’Donnell K, et al. Cohort Profile: Perinatal depression and child socioemotional development; the Bachpan cohort study from rural Pakistan. BMJ Open. 2019;9(5):e025644. doi: 10.1136/bmjopen-2018-025644 31061029 PMC6502044

[39] Sikander S, Ahmad I, Atif N, Zaidi A, Vanobberghen F, Weiss HA, et al. Delivering the Thinking Healthy Programme for perinatal depression through volunteer peers: a cluster randomised controlled trial in Pakistan. Lancet Psychiatry. 2019 Feb;6(2):128–39. doi: 10.1016/S2215-0366(18)30467-X 30686386

[40] Gallis JA, Maselko J, O’Donnell K, Song K, Saqib K, Turner EL, et al. Criterion-related validity and reliability of the Urdu version of the patient health questionnaire in a sample of community-based pregnant women in Pakistan. PeerJ. 2018;6:e5185. doi: 10.7717/peerj.5185 30038858 PMC6054083

[41] World Health Organization. Adverse Childhood Experiences International Questionnaire (ACE-IQ) [Internet]. 2018. Available from: https://www.who.int/violence_injury_prevention/violence/activities/adverse_childhood_experiences/en/.

[42] Kidman R, Smith D, Piccolo LR, Kohler HP. Psychometric evaluation of the Adverse Childhood Experience International Questionnaire (ACE-IQ) in Malawian adolescents. Child Abuse Negl. 2019 Jun;92:139–45. doi: 10.1016/j.chiabu.2019.03.015 30974257 PMC6513701

[43] Kazeem OT. A Validation of the Adverse Childhood Experiences Scale in Nigeria. Sex Abuse. 2015;7.

[44] Felitti VJ, Anda RF, Nordenberg D, Williamson DF, Spitz AM, Edwards V, et al. Relationship of Childhood Abuse and Household Dysfunction to Many of the Leading Causes of Death in Adults: The Adverse Childhood Experiences (ACE) Study. Am J Prev Med. 1998 May 1;14(4):245–58. 9635069 10.1016/s0749-3797(98)00017-8

[45] WHO Multicentre Growth Reference Study Group. WHO Child Growth Standards based on length/height, weight and age. Acta Paediatr Suppl. 2006 Apr;450:76–85. doi: 10.1111/j.1651-2227.2006.tb02378.x 16817681

[46] Bayley N. Bayley Scales of Infant Development-III. Psychological Corporation; 2006.

[47] Pendergast LL, Schaefer BA, Murray-Kolb LE, Svensen E, Shrestha R, Rasheed MA, et al. Assessing development across cultures: Invariance of the Bayley-III Scales Across Seven International MAL-ED sites. Sch Psychol Q. 2018 Dec;33(4):604–14. doi: 10.1037/spq0000264 30507236

[48] Ranjitkar S, Kvestad I, Strand TA, Ulak M, Shrestha M, Chandyo RK, et al. Acceptability and reliability of the bayley scales of infant and toddler development-III among children in Bhaktapur, Nepal. Front Psychol. 2018;9:1265. doi: 10.3389/fpsyg.2018.01265 30087639 PMC6066572

[49] Squires J, Bricker D. Ages & stages questionnaires, (ASQ-3). Parent-Complet Child Monit Syst 3rd Ed Baltim MD Brookes. 2009;

[50] Goodman R. The Strengths and Difficulties Questionnaire: a research note. J Child Psychol Psychiatry. 1997 Jul;38(5):581–6. doi: 10.1111/j.1469-7610.1997.tb01545.x 9255702

[51] Velikonja T, Edbrooke-Childs J, Calderon A, Sleed M, Brown A, Deighton J. The psychometric properties of the Ages & Stages Questionnaires for ages 2–2.5: a systematic review. Child Care Health Dev. 2017 Jan;43(1):1–17. 27554865 10.1111/cch.12397

[52] Samad L, Hollis C, Prince M, Goodman R. Child and adolescent psychopathology in a developing country: testing the validity of the strengths and difficulties questionnaire (Urdu version). Int J Methods Psychiatr Res. 2005;14(3):158–66. doi: 10.1002/mpr.3 16389892 PMC6878532

[53] Glymour MM, Greenland S. Causal diagrams. In: Modern Epidemiology. Third. Philadelphia, PA: Lippincott Williams & Wilkins; 2008.

[54] Shrier I, Platt RW. Reducing bias through directed acyclic graphs. BMC Med Res Methodol. 2008;8:1–15. 18973665 10.1186/1471-2288-8-70 PMC2601045

[55] Schisterman EF, Cole SR, Platt RW. Overadjustment bias and unnecessary adjustment in epidemiologic studies. Epidemiol Camb Mass. 2009 Jul;20(4):488–95. doi: 10.1097/EDE.0b013e3181a819a1 19525685 PMC2744485

[56] Seaman SR, White IR. Review of inverse probability weighting for dealing with missing data. Stat Methods Med Res. 2013 Jun;22(3):278–95. doi: 10.1177/0962280210395740 21220355

[57] Brookhart MA, Schneeweiss S, Rothman KJ, Glynn RJ, Avorn J, Sturmer T. Variable selection for propensity score models. Am J Epidemiol. 2006 Jun 15;163(12):1149–56. doi: 10.1093/aje/kwj149 16624967 PMC1513192

[58] Dugoff EH, Schuler M, Stuart EA. Generalizing observational study results: applying propensity score methods to complex surveys. Health Serv Res. 2014 Feb;49(1):284–303. doi: 10.1111/1475-6773.12090 23855598 PMC3894255

[59] Solberg MA, Peters RM. Adverse Childhood Experiences in Non-Westernized Nations: Implications for Immigrant and Refugee Health. J Immigr Minor Health. 2020 Feb;22(1):145–55. doi: 10.1007/s10903-019-00953-y 31811614

[60] Bokhari M, Badar M, Naseer U, Waheed A, Safdar F. Adverse Childhood Experiences & Impulsivity in Late Adolescence & Young Adulthood of Students of University of the Punjab Lahore. 2015;14.

[61] Pradeilles R, Norris T, Ferguson E, Gazdar H, Mazhar S, Bux Mallah H, et al. Factors associated with catch-up growth in early infancy in rural Pakistan: A longitudinal analysis of the women’s work and nutrition study. Matern Child Nutr. 2019;15(2):e12733. doi: 10.1111/mcn.12733 30345717 PMC6587826

[62] Engle PL, Black MM. The effect of poverty on child development and educational outcomes. Ann N Acad Sci. 2008;1136:243–56. doi: 10.1196/annals.1425.023 18579886

[63] Brown AL, McKenna BG, Cohen MF, Dunlop AL, Corwin EJ, Brennan PA. Maternal childhood adversity and early parenting: Implications for infant neurodevelopment. Int Public Health J. 2018;10(4):445–54.

[64] Bjorklund DF, Ellis BJ. Children, childhood, and development in evolutionary perspective. Dev Rev. 2014 Sep 1;34(3):225–64.

[65] Quinlan RJ. Human parental effort and environmental risk. Proc R Soc B Biol Sci. 2007 Jan 7;274(1606):121–5. doi: 10.1098/rspb.2006.3690 17134996 PMC1679876

[66] Rowell T, Neal-Barnett A. A Systematic Review of the Effect of Parental Adverse Childhood Experiences on Parenting and Child Psychopathology. J Child Adolesc Trauma. 2022 Mar 1;15(1):167–80. doi: 10.1007/s40653-021-00400-x 35222782 PMC8837768

[67] Tedeschi RG, Park CL, Calhoun LG. Posttraumatic growth: Positive changes in the aftermath of crisis. Routledge; 1998.

[68] Tranter H, Brooks M, Khan R. Emotional resilience and event centrality mediate posttraumatic growth following adverse childhood experiences. Psychol Trauma Theory Res Pract Policy. 2021;13(2):165–73. doi: 10.1037/tra0000953 32881570

[69] Greenberg DM, Baron-Cohen S, Rosenberg N, Fonagy P, Rentfrow PJ. Elevated empathy in adults following childhood trauma. PLoS One. 2018;13(10):e0203886. doi: 10.1371/journal.pone.0203886 30281628 PMC6169872

[70] Staub E, Vollhardt J. Altruism born of suffering: The roots of caring and helping after victimization and other trauma. Am J Orthopsychiatry. 2008;78(3):267–80. doi: 10.1037/a0014223 19123746

[71] Vollhardt JR. Altruism Born of Suffering and Prosocial Behavior Following Adverse Life Events: A Review and Conceptualization. Soc Justice Res. 2009 Mar 1; 22(1):53–97.

[72] Lim D, DeSteno D. Suffering and compassion: The links among adverse life experiences, empathy, compassion, and prosocial behavior. Emotion. 2016;16(2):175–82. doi: 10.1037/emo0000144 26751630

[73] Cooke JE, Racine N, Pador P, Madigan S. Maternal Adverse Childhood Experiences and Child Behavior Problems: A Systematic Review. Pediatrics [Internet]. 2021 Sep 1 [cited 2021 Sep 12];148(3). Available from: http://pediatrics.aappublications.org/content/148/3/e2020044131. doi: 10.1542/peds.2020-044131 34413250

[74] Monk C, Lugo-Candelas C, Trumpff C. Prenatal Developmental Origins of Future Psychopathology: Mechanisms and Pathways. Annu Rev Clin Psychol. 2019 May 7;15:317–44. doi: 10.1146/annurev-clinpsy-050718-095539 30795695 PMC7027196

[75] Thomas JC, Letourneau N, Campbell TS, Giesbrecht GF, Apron Study Team. Social buffering of the maternal and infant HPA axes: Mediation and moderation in the intergenerational transmission of adverse childhood experiences. Dev Psychopathol. 2018 Aug;30(3):921–39. 30068422 10.1017/S0954579418000512

[76] Racine N, Devereaux C, Cooke JE, Eirich R, Zhu J, Madigan S. Adverse childhood experiences and maternal anxiety and depression: a meta-analysis. BMC Psychiatry. 2021 Jan 11;21(1):28. doi: 10.1186/s12888-020-03017-w 33430822 PMC7802164

[77] Racine N, McDonald S, Chaput K, Tough S, Madigan S. Pathways from Maternal Adverse Childhood Experiences to Substance Use in Pregnancy: Findings from the All Our Families Cohort. J Womens Health 2002. 2021 Feb 1. doi: 10.1089/jwh.2020.8632 33524303

[78] Dunkel Schetter C. Psychological Science on Pregnancy: Stress Processes, Biopsychosocial Models, and Emerging Research Issues. Annu Rev Psychol. 2011 Jan 10;62(1):531–58. doi: 10.1146/annurev.psych.031809.130727 21126184

[79] Herba CM, Glover V, Ramchandani PG, Rondon MB. Maternal depression and mental health in early childhood: an examination of underlying mechanisms in low-income and middle-income countries. Lancet Psychiatry. 2016;3(10):983–92. doi: 10.1016/S2215-0366(16)30148-1 27650772

[80] McLaughlin KA, Sheridan MA, Lambert HK. Childhood Adversity and Neural Development: Deprivation and Threat as Distinct Dimensions of Early Experience. Neurosci Biobehav Rev. 2014 Nov;47:578–91. doi: 10.1016/j.neubiorev.2014.10.012 25454359 PMC4308474

[81] Machlin L, Miller AB, Snyder J, McLaughlin KA, Sheridan MA. Differential Associations of Deprivation and Threat With Cognitive Control and Fear Conditioning in Early Childhood. Front Behav Neurosci. 2019;13:80. doi: 10.3389/fnbeh.2019.00080 31133828 PMC6517554

[82] Greene CA, McCoach DB, Briggs-Gowan MJ, Grasso DJ. Associations among childhood threat and deprivation experiences, emotion dysregulation, and mental health in pregnant women. Psychol Trauma Theory Res Pract Policy. 2021 May;13(4):446–56. doi: 10.1037/tra0001013 33475412 PMC8217136

[83] Johnson D, Policelli J, Li M, Dharamsi A, Hu Q, Sheridan MA, et al. Associations of Early-Life Threat and Deprivation With Executive Functioning in Childhood and Adolescence: A Systematic Review and Meta-analysis. JAMA Pediatr. 2021 Jul 26;e212511. doi: 10.1001/jamapediatrics.2021.2511 34309651 PMC8314173

[84] Narayan AJ, Lieberman AF, Masten AS. Intergenerational transmission and prevention of adverse childhood experiences (ACEs). Clin Psychol Rev. 2021 Apr 1;85:101997. doi: 10.1016/j.cpr.2021.101997 33689982

[85] Bellis MA, Hughes K, Ford K, Hardcastle KA, Sharp CA, Wood S, et al. Adverse childhood experiences and sources of childhood resilience: a retrospective study of their combined relationships with child health and educational attendance. BMC Public Health. 2018;18(1):1–12. doi: 10.1186/s12889-018-5699-8 29940920 PMC6020215

[86] Bethell C, Jones J, Gombojav N, Linkenbach J, Sege R. Positive childhood experiences and adult mental and relational health in a statewide sample: Associations across adverse childhood experiences levels. JAMA Pediatr. 2019;173(11):e193007–e193007. 10.1001/jamapediatrics.2019.3007 PMC6735495 31498386

[87] Crouch E, Radcliff E, Strompolis M, Srivastav A. Safe, stable, and nurtured: Protective factors against poor physical and mental health outcomes following exposure to adverse childhood experiences (ACEs). J Child Adolesc Trauma. 2019;12(2):165–73. doi: 10.1007/s40653-018-0217-9 32318189 PMC7163854

[88] Morris AS, Hays-Grudo J, Zapata MI, Treat A, Kerr KL. Adverse and Protective Childhood Experiences and Parenting Attitudes: the Role of Cumulative Protection in Understanding Resilience. Advers Resil Sci. 2021 Sep 1;2(3):181–92. doi: 10.1007/s42844-021-00036-8 33778769 PMC7987739

[89] Floyd K, Morman MT. Affection received from fathers as a predictor of men’s affection with their own sons: Tests of the modeling and compensation hypotheses. Commun Monogr. 2000 Dec 1;67(4):347–61.

[90] LeMasters K, Bates LM, Chung EO, Gallis JA, Hagaman A, Scherer E, et al. Adverse childhood experiences and depression among women in rural Pakistan. BMC Public Health. 2021;21(1):1–11. 33632175 10.1186/s12889-021-10409-4 PMC7905421

