# Supplementary material for: Maternal adverse childhood experiences on child growth and development in rural Pakistan: An observational cohort study
Source: PLOS Glob Public Health. 2023 Oct 25;3(10):e0001669. doi: 10.1371/journal.pgph.0001669 (PMC10599588; doi:10.1371/journal.pgph.0001669)
Supplement: S1 Fig — (DOCX) [file pgph.0001669.s001.docx]

| 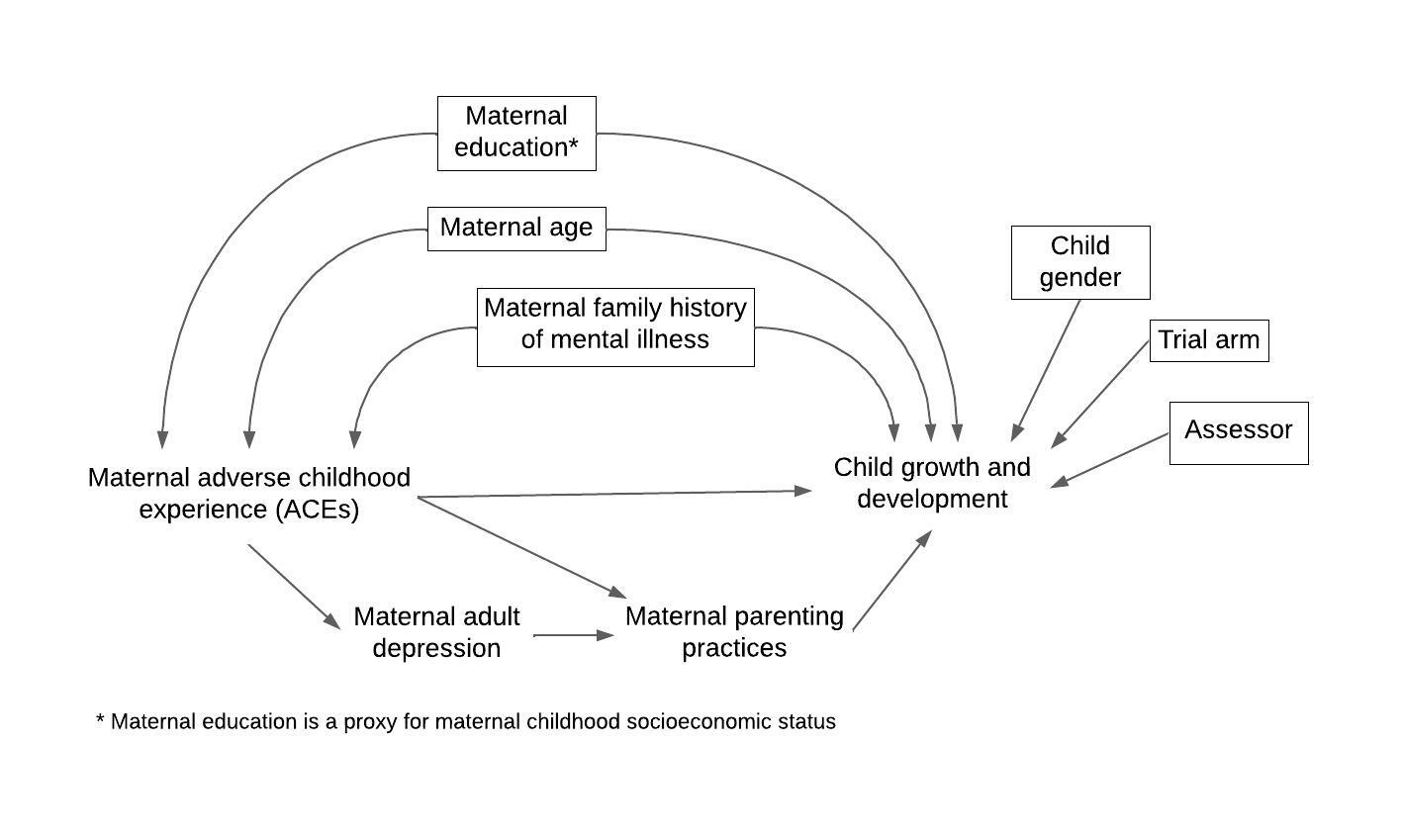 |
| --- |
| **S1 Figure. Maternal ACEs and child development directed acyclic graph.**  We used a directed acyclic graph (DAG) to identify potential confounders and mediators of the relationship between maternal ACEs and child growth and development. Based on the DAG, we only included maternal age, education, and family history of mental illness as confounders. Maternal adult depression and parenting practices were identified as mediators; therefore, we did not include them in our models. We included child gender, trial arm, and assessor in the models to improve precision. |
