# Supplementary material for: Maternal adverse childhood experiences on child growth and development in rural Pakistan: An observational cohort study
Source: PLOS Glob Public Health. 2023 Oct 25;3(10):e0001669. doi: 10.1371/journal.pgph.0001669 (PMC10599588; doi:10.1371/journal.pgph.0001669)
Supplement: S2 Table — (DOCX) [file pgph.0001669.s003.docx]

| **S2 Table. Maternal ACEs and child growth, Bachpan Cohort, Pakistan (n=877)** | | | |
| --- | --- | --- | --- |
|  | **LAZ** | **WAZ** | **WLZ** |
|  | **β (95% CI), CLD** | | |
| *Total score* | -0.04 | 0.01 | 0.04 |
|  | (-0.09 - 0.01), 0.10 | (-0.04 - 0.05), 0.09 | (-0.01 - 0.09), 0.10 |
|  | **MD (95% CI), CLD** | | |
| *Any vs. none* | -0.12 | 0.02 | 0.14 |
|  | (-0.30 - 0.05), 0.35 | (-0.13 - 0.17), 0.30 | (-0.03 - 0.31), 0.34 |
|  |  |  |  |
| *ACE categorical* |  |  |  |
| None | *ref* | *ref* | *ref* |
| One | -0.10 | 0.02 | 0.12 |
|  | (-0.34 - 0.14), 0.48 | (-0.18 - 0.21), 0.39 | (-0.09 - 0.33), 0.42 |
| Two | -0.12 | 0.09 | 0.24 |
|  | (-0.37 - 0.12), 0.49 | (-0.12 - 0.29), 0.41 | (-0.00 - 0.47), 0.47 |
| Three | -0.13 | -0.08 | -0.00 |
|  | (-0.33 - 0.08), 0.41 | (-0.30 - 0.14), 0.44 | (-0.31 - 0.30), 0.61 |
| Four or more | -0.20 | 0.03 | 0.20 |
|  | (-0.48 - 0.09), 0.57 | (-0.19 - 0.25), 0.44 | (-0.08 - 0.48), 0.56 |
| *ACE Domains* |  |  |  |
| Neglect | 0.17 | 0.23 | 0.21 |
|  | (-0.23 - 0.56), 0.79 | (-0.07 - 0.53), 0.60 | (-0.02 - 0.44), 0.46 |
| Family psychological distress | -0.15 | -0.07 | 0.01 |
|  | (-0.32 - 0.03), 0.35 | (-0.26 - 0.11), 0.37 | (-0.19 - 0.22), 0.41 |
| Home violence | -0.09 | -0.04 | 0.01 |
|  | (-0.31 - 0.12), 0.43 | (-0.22 - 0.13), 0.35 | (-0.14 - 0.16), 0.30 |
| Community violence | -0.10 | 0.09 | 0.21 |
|  | (-0.40 - 0.21), 0.61 | (-0.16 - 0.34), 0.50 | (-0.07 - 0.50), 0.57 |
| We present β estimates for the Total score and mean differences for all other operationalizations. 95% confidence intervals and confidence limit differences are also shown. We used weighted generalized linear models with cluster robust standard errors. Sampling and inverse probability censoring weights were combined. All models controlled for baseline maternal age, maternal education, trial arm, assessor, and child gender.  Abbreviations: Length-for-age z-score (LAZ); Weight-for-age z-score (WAZ); Weight-for-length z-score (WLZ); Confidence limit difference (CLD); Mean difference (MD) | | | |
|  |  |  |  |
|  |  |  |  |
